# Sol–Gel Photonic Glasses: From Material to Application †

**DOI:** 10.3390/ma16072724

**Published:** 2023-03-29

**Authors:** Giancarlo C. Righini, Cristina Armellini, Maurizio Ferrari, Alice Carlotto, Alessandro Carpentiero, Andrea Chiappini, Alessandro Chiasera, Anna Lukowiak, Thi Ngoc Lam Tran, Stefano Varas

**Affiliations:** 1 Nello Carrara Institute of Applied Physics (IFAC-CNR), MiPLab, Via Madonna del Piano 10, 50019 Sesto Fiorentino, Italy; 2 IFN-CNR CSMFO Laboratory and FBK Photonics Unit, Via alla Cascata 56/C Povo, 38123 Trento, Italymaurizio.ferrari@ifn.cnr.it (M.F.); alice.carlotto@ifn.cnr.it (A.C.); alessandro.carpentiero@ifn.cnr.it (A.C.); andrea.chiappini@ifn.cnr.it (A.C.); alessandro.chiasera@ifn.cnr.it (A.C.); lam.tran@ifn.cnr.it (T.N.L.T.); stefano.varas@ifn.cnr.it (S.V.); 3 Institute of Low Temperature and Structure Research, PAS, ul. Okólna 2, 50422 Wroclaw, Poland; a.lukowiak@intibs.pl; 4 Department of Physics, Politecnico di Milano, Piazza Leonardo da Vinci 32, 20133 Milano, Italy; 5 Department of Materials Technology, Faculty of Applied Science, Ho Chi Minh City University of Technology and Education, Vo Van Ngan Street 1, Thu Duc District, Ho Chi Minh City 720214, Vietnam

**Keywords:** glass photonics, sol–gel systems, rare-earth luminescence, optical waveguides, integrated optics, microresonators, metastructures, opals

## Abstract

In this review, we present a short overview of the development of sol–gel glasses for application in the field of photonics, with a focus on some of the most interesting results obtained by our group and collaborators in that area. Our main attention is devoted to silicate glasses of different compositions, which are characterized by specific optical and spectroscopic properties for various applications, ranging from luminescent systems to light-confining structures and memristors. In particular, the roles of rare-earth doping, matrix composition, the densification process and the fabrication protocol on the structural, optical and spectroscopic properties of the developed photonic systems are discussed through appropriate examples. Some achievements in the fabrication of oxide sol–gel optical waveguides and of micro- and nanostructures for the confinement of light are also briefly discussed.

## 1. Introduction

Glass is a unique material, due to the tunability of its physicochemical characteristics and the broadness of its application spectrum. It is not by chance that recently two colleagues from Corning Inc. underlined that the world has entered the Glass Age [1]. We feel that everyone should share their thought: “We have an unprecedented opportunity to harness the unique capabilities of glass to solve some of our world’s most urgent challenges, such as more effective healthcare, cleaner energy and water, and more efficient communication”. It was also on such a base that the General Assembly of the United Nations unanimously decided to declare 2022 the International Year of Glass (IYoG); a multi-authored book, which was printed for the IYoG opening ceremony in Geneva, vividly illustrated some fundamental aspects of the history and technology of glass, also from the point of view of education and sustainability [2].

Under the glass name, one may encounter many families, whose properties have been exploited in very different application areas, from tableware to architecture, from spectacles to telescopes, from biomedicine to optical communications. Among such families, oxide glasses play a leading role, and silica-based glasses constitute a group of materials fundamental in many scientific implementations. The use of silicate glasses is consolidated in a broad spectrum of applications, most of them crucial for the way of life to which we are accustomed nowadays [3]. Referring in particular to the photonics field, we owe to ultrapure silica the development of ultra-low-loss optical fibers: the record value of 0.1419 dB/Km at 1560 nm wavelength, very close to the theoretical limit, was obtained with a slightly fluorine-doped silica core surrounded by a fluorine-doped cladding [4]. This has later led to the development of silica-based platforms for integrated photonics, such as the low-loss, low-cost silica-titania platform [5]. The activation of silicate glasses with rare-earth (RE) ions has been another groundbreaking advance on which much glass photonics is built [6]. 

The synthesis of silicate glasses is often performed by the traditional melting process; the need for glass films for optical and integrated photonic applications, however, has pushed the development of optimized thin film deposition methods (radio-frequency sputtering, chemical vapor deposition (CVD), pulsed-laser deposition, et cetera) [7]. Starting in the early 1980s, another route has gained much interest, based on sol–gel technology, which represents a low-temperature, low-cost wet chemistry approach. Sol–gel is now widely followed as an effective alternative for synthesizing both bulk and thin-film glasses, and keeps attracting continuously growing scientific and manufacturing attention. The very first steps in sol–gel chemistry were made in 1846 by Ebelmen [8], who observed the formation of a glass-like material due to the hydrolysis of silica alkoxides; however, the terms hydrosol and hydrogel of silicic acid, and the corresponding alcosols and alcogels were introduced only in 1846 by Graham [9]. Real advances may be dated to 1919, when Patrick patented a process to produce silica gel in large quantities from sodium silicate (Na_2_SiO_3_) [10]. In the field of optics, in 1939, Geffcken and Berger from Schott Glaswerke Company patented a process, based on the sol–gel method, to make single-oxide coatings [11], but only in 1953 did the first products appear on the market [12].

In the late 1960s, the concept of integrated optics, based on light confinement in transparent thin films, came out at Bell Labs in the US [13], and soon afterwards the importance of solution-deposited films for planar waveguides was outlined by some of Miller’s fellow researchers [14]. The last fifty years have seen sol–gel strongly contributing to the development of low-loss high-quality passive and active optical waveguides, but also of special optical coatings, photonic crystals, phosphors, and multifunctional materials [15].

**Figure 1:**
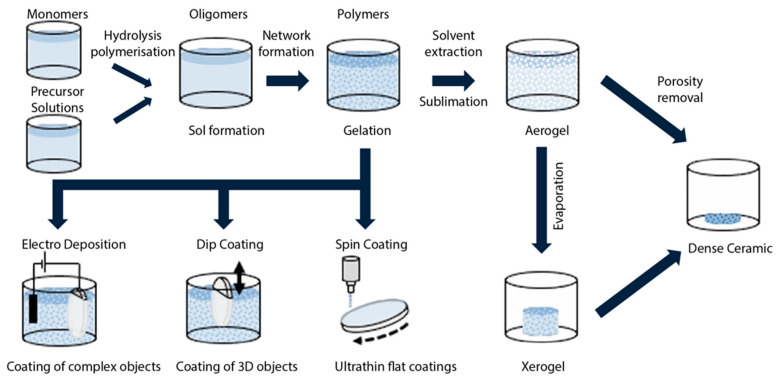
Simplified illustration of the main steps in the fabrication of bulk or thin-film sol–gel glasses or ceramics. A specific additional heat treatment may lead to the creation of glass-ceramics. Aerogel and xerogel are two forms of dried gels that can retain their porous texture after the drying process; aerogels are less dense and have a larger surface area and porosity (the porosity, namely the non-solid volume, must be greater than 50%). Either one is obtained from a gel depending on the drying rate. Aerogels find large application in thermal insulation, adsorption, catalysis, drug delivery systems, and aerospace. Reproduced with modifications from [16] under a Creative Commons license.

Figure 1 tries to summarize the fundamental steps of the sol–gel process to create bulk or thin-film glasses, ceramics and glass-ceramics. The process steps indicated in the figure may well be altered, extended or canceled, depending on the final goal. The thermal treatments at the various stages are also critical and vary as a function of the desired result. The deposition stages, dip coating and spin coating in particular, are of the greatest importance for sol–gel photonic applications.

Going back to the general area of sol–gel physics and chemistry, the 1980s saw the publication of only 172 papers, but in the following decade (1990–2000) the number of publications rose to 2950 and today the cumulative number is greater than 19,000 [17].

Sol–gel synthesis has proven to be particularly useful in the case of photonic glasses, where several issues must be faced, in particular, concerning the transparency and the composition, the latter being governed by the phase diagram. Sol–gel technology allows us to partially overcome such a constraint and also to improve the rare-earth solubility. The critical problem of luminescence quenching due to the physical clustering of rare-earth ions can be strongly mitigated by the appropriate composition and densification protocols, when the sol–gel route is employed [18]. Moreover, sol–gel technology allows us to develop photonic glasses with peculiar compositions and properties, in different forms (bulks, nanoparticles or thin films) and with significant characteristics for different photonic applications. Some 340 papers have been published so far in this area [19], starting with the pioneer paper by Dunn and Zink, in 1991, who concluded that organic-doped sol–gel materials were emerging as an important means of producing photonic materials [20]. It is interesting to note that an earlier paper, published by Ramaswamy et al., in 1988 [21], reported the higher optical transmission (measured attenuation 0.21 ± 0.02 dB/cm) and lower dispersion of a silica sol–gel bulk glass compared to commercial silica; two routes were suggested for fabricating optical waveguides in such bulk glass; that is, ion diffusion (they attempted to diffuse barium, but the results were not satisfactory) or local heating to increase the density and index of the glass. Later, a good review of early works on sol–gel photonic glasses was presented in the papers’ selection edited by Najafi, in 1998 [22], whereas an overview of more current research is provided in the already mentioned book edited by Almeida et al. [15].

The present paper provides a short survey of some aspects crucial in sol–gel photonics that we have faced in our experimental activity and that could have some utility for both expert and early-stage researchers who may be fascinated by the sol–gel route in optics. The general focus is on the sol–gel synthesis and the characterization of rare-earth-doped oxide glasses and the optical waveguides produced therein. After this introduction, some early research is recalled, that puts in evidence the challenges of the first approaches to sol–gel photonics. The attention is focused on the densification process, the luminescence quenching and the experimental techniques to study the related mechanisms. The third section presents a summary of relevant experimental results achieved in the field of optical waveguides, and the fourth section discusses some more recent results regarding the functionalization of micro/nanostructures such as photonic crystals and microresonators using the sol–gel approach.

## 2. Sol–gel-Derived Silicate Glasses for Photonics

**Figure 2:**
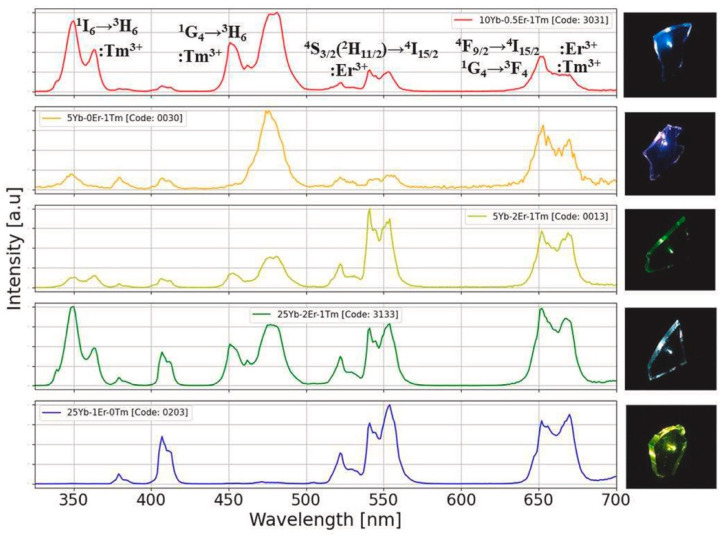
UV–VIS emission spectra by excitation at 980 nm of nano-glass-ceramics with composition 95SiO_2_–5NaYF_4_ and different co-doping concentrations of Yb, Er and Tm ions. The corresponding images of upconversion light from the sol–gel samples are shown in the right-hand column. The upper plot also indicates the electronic transitions of rare-earth ions giving rise to the main upconversion emission bands. Reproduced from [29] under a Creative Commons license.

An important step forward in sol–gel photonics was made when, pushed by the development of optical amplifiers in fibers, researchers started to investigate silica xerogels activated by rare-earth ions with the aim to obtain compact structures with the same transparency as silica glasses produced by melting but with more tailored properties and functionalities [18,23]. Rare-earth-doped sol–gel materials find many other applications than in the light amplification and optical communications areas, such as, for instance, lighting [24], smart windows [25], energy conversion and solar cells [26,27,28] and anti-counterfeiting [29]. Referring to the last application, luminescence tags with photonic materials have recently been worthy of great attention: the codification of the luminescence pattern and/or the encryption of a digital code based on the intensity ratios of the resulting spectra represent two ways of achieving effective anti-counterfeiting procedures. As an example, Figure 2 shows, on the left, the upconversion emission spectra, by excitation at 980 nm, of some sol–gel glass-ceramic samples of 95SiO_2_–5NaYF_4_ composition, co-doped with different concentrations of Yb, Er and Tm ions [29]. The four-digit code which appears in the plots is generated by the luminescence intensity ratios among the upconversion bands, at around 350, 480, 550 and 660 nm. For each spectrum, the intensities are normalized to their corresponding maximum.

As a general rule, the sol–gel technique exhibits noteworthy advantages with respect to other glass-synthesis methods: it allows us to overcome the constraints imposed by the phase diagram, the fabrication procedure strongly reduces energy consumption, and it is possible to obtain glasses of unconventional shape. The final properties of a sample, however, are strongly dependent on the full physicochemical process, namely, thermal treatments, dehydration and sintering steps. In particular, the negative effect of residual water on the spectroscopical properties of the RE ions embedded in the silica matrix is well known [30,31]. Therefore, the study of the modification of a sol–gel system as a function of its densification and thermal history is very important [32,33].

One of the first papers focusing on this matter in the case of sol–gel photonics concerned the investigation of Eu^3+^-doped silica glasses produced by the sol–gel method and heat-treated in the temperature range T = 80–1100 °C [32]. The authors performed Raman and photoluminescence (PL) measurements on the xerogel samples and concluded that the intensities of the bands at 3750 cm^−1^, assigned to OH stretching, and 980 and 490 cm^−1^, assigned to Si-OH bond, were decreasing with the increasing of T, but hydroxyl and organic groups were present even in the sample treated at 900 °C. Measurements of PL lifetimes revealed the presence of non-radiative relaxation due to OH stretching vibrations even in samples with higher densification. A complementary characterization of the xerogels as a function of the densification was performed by Campostrini et al. [33] by means of thermogravimetric analysis. The measurements revealed a weight loss of 12% in the range 60–265 °C, corresponding to the stripping of the solvent entrapped in the pores; another 4% loss in the range 265–1000 °C was related to the removal of residual ethoxyl, methyl and hydroxyl groups. IR absorption measurements evidenced complete densification at 1200 °C with the disappearance of the typical band of the hydroxyl groups. Again, large inhomogeneities in the lifetimes were found, indicating that, even at the higher temperatures, still OH groups were retained by the system and only a fraction of the Eu^3+^ ions were far from hydroxyl groups and had decay with their characteristic radiative lifetime. Lately, Bouajaj et al. [34] studied the ^5^D_0_→^7^F_0_ transition of the same system and verified that emission and absorption at low T were not resonant; this effect was attributed to a redistribution of the excitation energy among the different environments of the RE ions through the energy transfer process. The experimental emission spectra were also compared with simulated ones and a good agreement was obtained.

**Figure 3:**
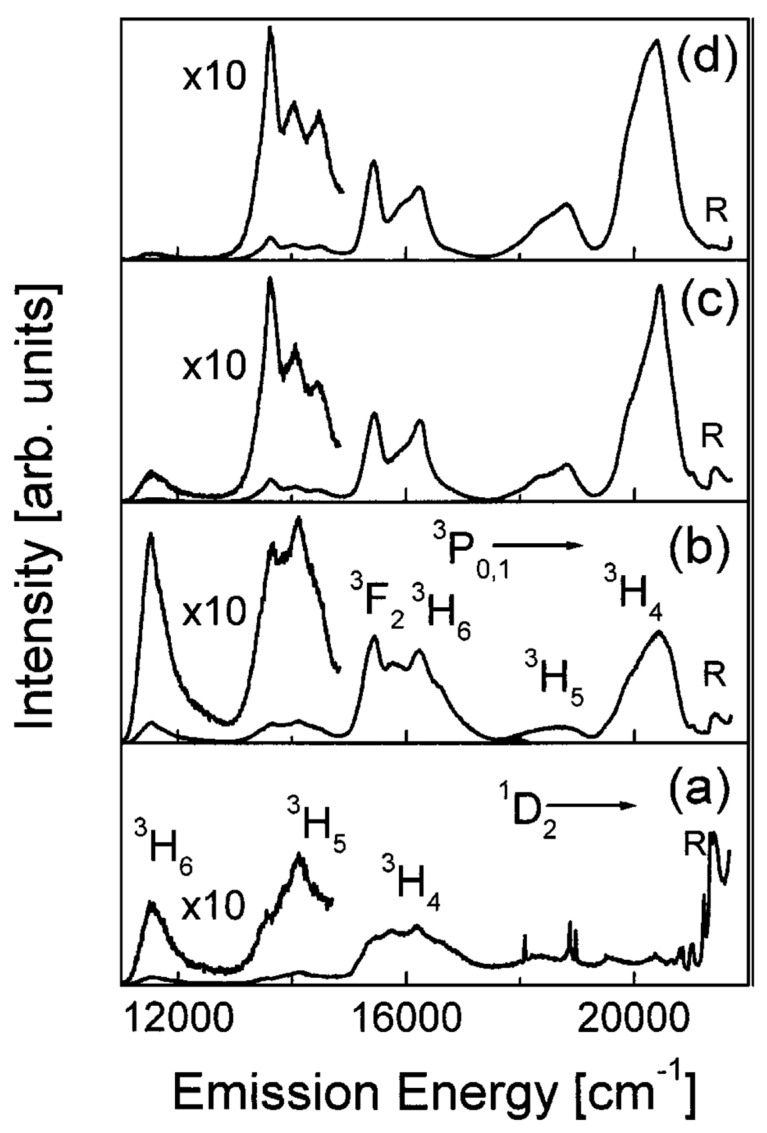
Room-temperature (RT) luminescence spectra of the 500 (**a**), 10,000 (**b**), 20,000 (**c**), and 100,000 (**d**) Pr/Si ppm doped silica xerogels. Excitation was at 457.9 nm. The main emissions from the ^3^P_0; 1_ and ^1^D_2_ states are indicated. R labels the Raman band at 430 cm^−1^. Reproduced with permission from [35], copyright 1969, Kluwer Academic Publishers.

An interesting study on the effect of RE concentration on the number of OH groups in silica xerogels was reported by Armellini et al. [35]. There, SiO_2_ xerogels, with Pr^3+^ ion concentrations ranging from 200 to 100,000 ppm and treated for 120 h at 900 °C, were investigated by Fourier-transform infrared spectroscopy (FTIR), near-infrared (NIR) absorption, Raman and luminescence spectroscopies. For low-RE concentrations (from 200 up to 1000 ppm), the xerogels showed a higher content of OH groups, and only the emission from ^1^D_2_ state was observed; for higher Pr^3+^ concentrations, emissions occurred from both ^3^P_0_ and ^1^D_2_ states; while, for samples with more than 10,000 ppm, emissions were reduced due to the cross-relaxation process. Figure 3 shows the luminescence spectra obtained by exciting samples at 457.9 nm within the ^3^H_4_→^3^P_2_ absorption band of Pr^3+^. The silica samples were doped with (a) 500, (b) 10,000, (c) 20,000 and (d) 100,000 Pr/Si ppm, respectively.

**Figure 4:**
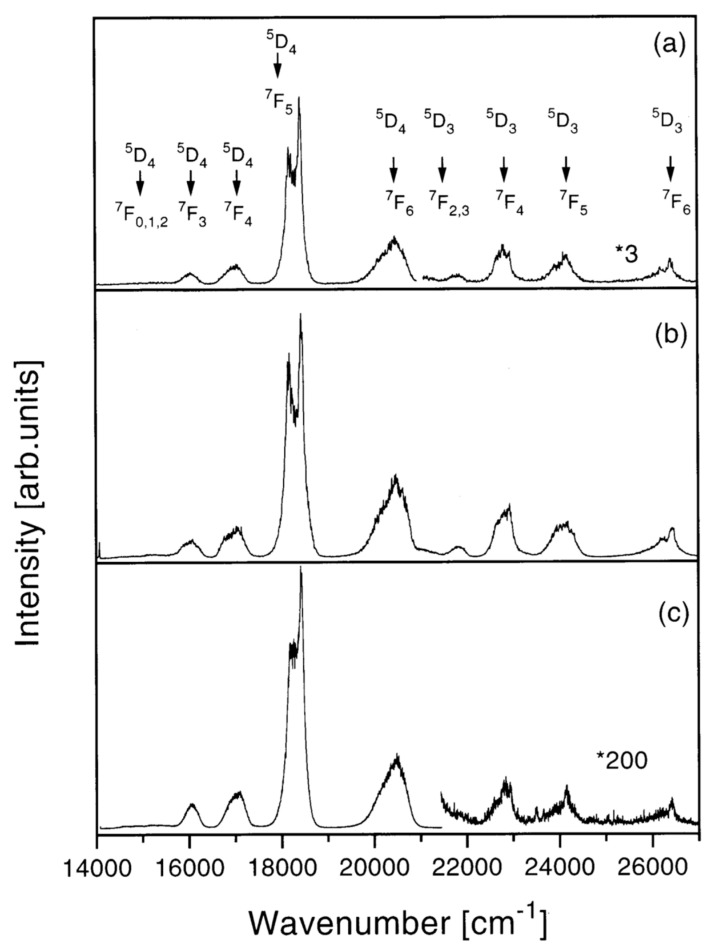
Room-temperature luminescence spectra after excitation at 355 nm of the silica xerogel samples: (**a**) 200 ppm (900 °C, 120 h); the right end of the spectrum is magnified 3 times (*3), (**b**) 400 ppm (900 °C, 72 h), and (**c**) 10,000 ppm (900 °C, 120 h) Tb:Si; the right part of the spectrum is magnified 200 times (*200). Reproduced with permission from [36], copyright 1998, Elsevier.

The clustering effect is another challenging issue in activated photonic glasses, mainly related to the large miscibility gap in the RE_2_O_3_–SiO_2_ system. The rare-earth clustering strongly reduces the luminescence quantum yield and there is a huge number of experimental and theoretical studies on this topic. One research paper, which investigated the problem to find appropriate solutions, dealt with Tb^3+^ ions in silica xerogels. Pucker et al. performed structural and optical characterizations of Tb^3+^-doped xerogels with concentrations ranging from 200 to 40,000 ppm [36]. Emission spectra and decay curve analysis evidenced that Tb^3+^ ions had a strong tendency to form clusters even at low concentrations; moreover, they could be found in two different locations: inside the clusters and well separated. Figure 4 shows the photoluminescence spectra obtained upon excitation at 355 nm of the samples doped with: (a) 200 ppm, (b) 400 ppm, (c) 10,000 ppm Tb^3+^, respectively. All the samples presented emissions from the ^5^D_4_ state. Emissions from the ^5^D_3_ state were observed in the samples containing fewer than 20,000 ppm of Tb^3+^ ions. The intensity ratio between ^5^D_3_ and ^5^D_4_ emissions was found to decrease with the increase in RE ion concentrations and the quenching of the ^5^D_3_ emissions was attributed to cross-relaxation processes between ions. The probability of these processes increases with the decrease in distance between donor and acceptor (in this case, both Tb^3+^ ions), as was confirmed by lifetime measurements. Moreover, the decay curve analysis demonstrated the tendency to also cluster formation for very low Tb^3+^ concentrations. In agreement with the OH vibration effect mentioned above, the luminescence of the ^5^D_3_ state was observed in densified samples only. In fact, the OH stretching vibrations induce effective non-radiative relaxation from the ^3^D_3_ level to the ^5^D_4_ state.

The importance of efficient light emission by RE-doped sol–gel bulk and thin-film glasses has promoted a large number of studies of the way to improve the fluorescence yield in these materials [37,38,39,40,41,42,43,44,45,46]. One of the ways to enhance fluorescence is based on aluminum co-doping; its positive effect on the fluorescence properties of RE-doped glasses [47], and in particular of sol–gel glasses [37,48,49,50] has been known for a long time, although the physical-chemical mechanism is still an object of research. One possible mechanism is suggested in this picture: when the aluminum ions are added, they may be incorporated in two local bonding configurations in the silica network, namely, a tetrahedral bonding configuration, such as AlO_4/2_ groups, as a network former, and an octahedral coordination of oxygen atoms, such as AlO_6/2_ groups, as a network modifier [37,47]. These groups could act as solvation shells in the glass network for the rare earth. In the case of the AlO_4/2_ groups, due to charge compensation, the trivalent rare-earth ions are preferentially accommodated near to the aluminum sites. In the network modifier case, the aluminum ions break the silica structure, producing non-bridging Al–O groups, which can coordinate the trivalent ions. A detailed discussion about the role of the solubility of the rare-earth ions in aluminum co-doped silica glasses is given in [6]. A recent work, studying Eu- and Tb-doped sol–gel glasses [41], provided new insights into the role of Al^3+^ co-doping in improving the fluorescence yield of glasses containing rare-earth (RE) ions. It was suggested that the fluorescence enhancement by Al co-doping was due to different mechanisms, depending on the Al:RE ratio: at low Al concentrations, the site symmetry is lowered, and the transition probability is increased; at a high Al:RE ratio, the highest frequency phonons are of lower energy and therefore nonradiative decay rates are reduced. In another recent study, dealing with Er^3+^/Al^3+^-doped silica glasses with Al/Er ratios from 0 to 200 [45], it was shown that, as the Al/Er ratio increased, the number of Al^3+^ ions around the Er^3+^ ions gradually increased, too. Concurrent structural changes led to the site-to-site variations in the erbium local environment and finally to the inhomogeneous broadening of the absorption and emission spectra, with a decrease in both absorption and emission at 1.53 µm. With the increase in Al/Er ratio, the full width at half maximum (FWHM) of the emission increased from 27.2 to 54.3 nm, suggesting a route to the optimized design of broadband amplifiers. 

Aluminum co-doping, however, is not the only effective tool to enhance photoluminescence: the use of drying control chemical additives during gel preparation [39,40] and the introduction of nanoparticles and nanocrystals [39,43,44,46] have proven to be among the other feasible routes to achieve higher emission intensities from RE-doped sol–gel glasses.

## 3. Sol–gel Optical Waveguides

As already mentioned, optical planar and channel waveguides in sol–gel silica-based glasses have been studied for a long time [14,21,22,23]. Besides thin film deposition, the fabrication of three-dimensional waveguides (channel, ridge, rib, etc.) requires classical photolithography or direct laser writing [51]. Following the early suggestion by Ramaswamy [21], laser densification and laser writing of ridge waveguides by a CO_2_ laser were demonstrated by some of us in the 1990s [52,53]. Later, fs-laser writing started to be used to fabricate 3D waveguides in sol–gel glasses [54,55,56,57] and soon became the most effective technique. An interesting review on sol–gel thin-film processing and patterning, with a focus on the application as integrated waveguide sensors, was recently published [58].

The most critical characteristic of optical waveguides for application in integrated photonics is constituted by propagation loss: the value of 1 dB/cm has been widely considered the upper acceptable limit. Many articles, however, report results with propagation losses higher than 1 dB/cm as preliminary experiments and/or under the claim that losses may later be reduced through optimized processing. Table 1 reports the values of propagation loss measured in some sol–gel waveguides with different compositions; they range from 0.06 to 1.5 dB/cm, approximately. The table does not aim to be exhaustive; on the contrary, it only serves the purpose of showing the various attempts pursued over more than 40 years of activity in this field. It must also be underlined that the values of loss cannot be truly compared to each other since they often refer to different structures, namely, different layer thicknesses, depositions made on different substrates, and, sometimes, even different claddings (air, another glass layer, etc.). Finally, the accuracy of the loss measurement was definitely not the same in all the examples; in some cases, no indication of measurement error was given.

**Figure 5:**
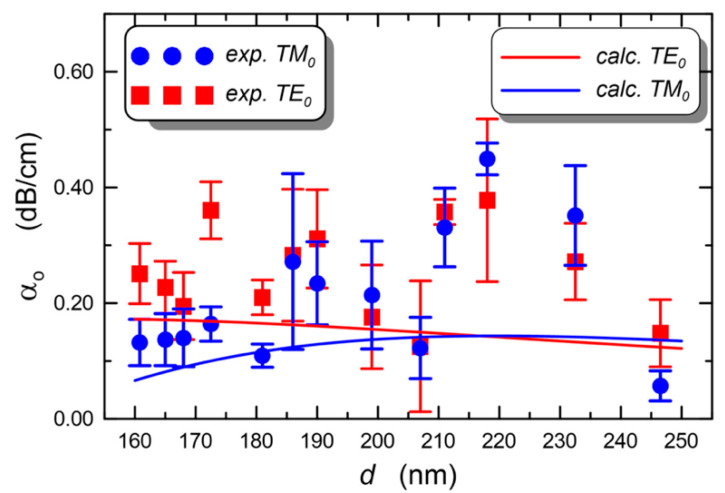
Experimental and calculated loss of fundamental TE and TM modes at 677 nm propagating in a silica-titania sol–gel single-mode waveguide deposited onto a BK7 substrate, as a function of the layer thickness. Reproduced from [59] under a Creative Commons license.

The application of silica-titania sol–gel films to evanescent wave sensors is the subject of another recent review, where an in-depth analysis of the sources of propagation loss in an optical waveguide is also presented [59]. Experimental tests of waveguide losses were made on a series of samples, all made of the same sol but using different speeds of BK7 substrate withdrawal; all samples were simultaneously annealed at 500 °C for 60 min. The resulting waveguides were all single-mode, with thicknesses in the range 160 to 246 nm. Figure 5 shows the behavior of the calculated and measured propagation loss as a function of the layer thickness. The lowest optical loss α_0_ = 0.06(3) dB/cm was measured for the TM_0_ mode in the layer with a thickness of d = 246 nm, while the highest TM_0_ loss (α_0_ = 0.15(6) dB/cm) was in the layer 218 nm thick. The loss of the fundamental transversal electric mode TE_0_ in the two layers was α_0_ = 0.45(3) dB/cm and 0.38(14) dB/cm, respectively [59].

**Table 1:** Propagation loss of various optical waveguides fabricated by sol–gel technique (vc indicates that experiments were made on different glass compositions; TMSPM: trimethoxysilylpropyl methacrylate).

Glass	Waveguide	*n*	Loss dB/cm	Wavelength µm	Note	Reference
Silica-titania (vc)	planar	1.6–1.9	<1.0	0.6328		[60]
Silica-titania (1:1)	planar	1.72	0.6	0.6328		[61]
Silica-titania Si-Ti-Al (vc)	planar	1.7–1.8	<0.5 average	0.6328	Aging effects	[62]
Silica-titania (vc)	slab	~1.8	0.06	0.677		[59]
Bulk silica xerogel	channel		2.9 0.7	0.6	2-line fs-micromachined	[57]
Silica-zirconia + photosensitizer (vc)	channel	1.48–1.52	0.1	1.55	UV exposure	[63]
Hybrid organic-inorganic	channel	1.502–1.562	0.5 1.5	1.310 1.550	Laser writing and etching	[64]
Hybrid silica-phenil groups	planar	~1.50–1.55	0.23	0.6328		[65]
Hybrid Si-Ce	slab	1.47	1.5	0.6328	Si, Ti, Zr, Ce	[66]
Zn-Si glass-ceramic	slab	1.529	1.4	0.6328		[67]
Tin-silica Eu^3+^ glass-ceramic	slab	1.557	0.5 ± 0.2	0.6328	Photorefractive	[68]
Hybrid Zr-doped TMSPM	slab and channel		0.16 (slab)	1.550	Photolithography	[69]

## 4. Sol–gel Derived Photonic Micro- and Nanostructures

Another interesting research activity in the field of sol–gel photonics is related to photonic systems at micro and nano scales. Here, we limit ourselves to consider two examples: artificial opals, or colloidal crystals, which are a type of three-dimensional photonic bandgap structure, and micro/nanospheres acting as optical resonators.

### 4.1. Three-Dimensional (3D) Photonic Crystals

Three-dimensional ordered photonic crystals may be fabricated on solid substrates from colloidal systems and the self-assembling of nanospheres [70,71]. As an example, the design and fabrication of photonic structures constituted by sol–gel-derived pure and Er^3+^-doped silica spheres were discussed in 2007 by Chiappini et al. [72]. Highly monodisperse SiO_2_ nanospheres with 270 nm diameter were synthesized by means of the Stöber method: tetraethyl orthosilicate (TEOS), water, and ammonia were used in the following concentrations: 0.22 M, 15 M and 1 M, respectively. Two mother solutions were prepared, the first one constituted by TEOS and ethanol (EtOH) and the second one by NH_3_, H_2_O and EtOH. They were mixed quickly and maintained under stirring at constant temperature and humidity for 24 h. The ammonia acts as catalyzer for the TEOS hydrolysis and condensation, and nanospheres are formed. The suspension was then washed with water by means of repeated centrifugation; finally, the separated SiO_2_ beads were dried at 80 °C.

**Figure 6:**
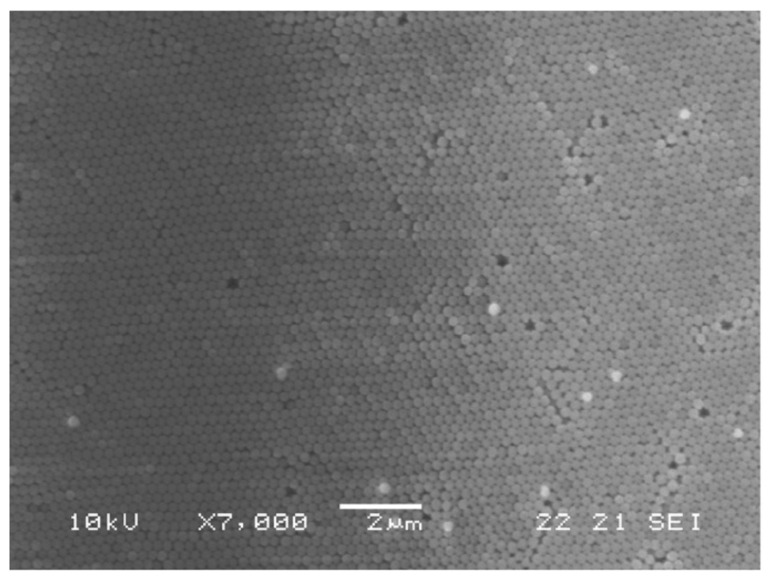
SEM micrograph of the top surface of the opal structure formed by the vertical deposition method of 270 nm diameter silica spheres. Reproduced with permission from [72], copyright 2007, Elsevier.

**Figure 7:**
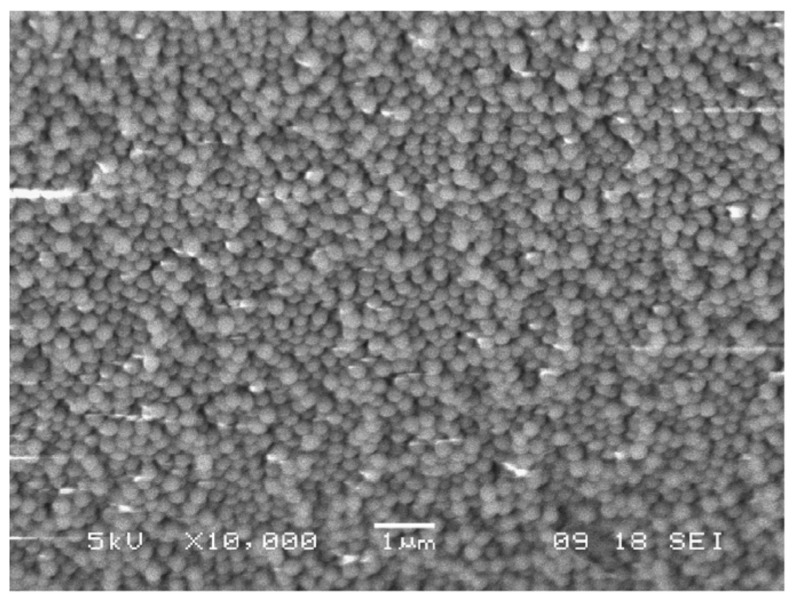
SEM image of the core-shell-like particles after seeded growth using the acid-based reaction. Particle size is again around 270 nm. Reproduced with permission from [72], copyright 2007, Elsevier.

To synthesize core-shell Er^3+^ doped silica spheres, the protocol was changed in order to work in an acidic environment, to avoid the precipitation of RE hydroxides in a basic pH environment. For this reason, previously prepared SiO_2_ spheres were coated with a Er^3+^-doped silica shell. In detail, 150 mg of SiO_2_ shells were introduced in a solution constituted by TEOS:CH_3_COOH:H_2_O with the molar ratio 1:8:8, where ErCl_3_ was added in the concentration of 0.2% with respect to SiO_2_. After 45 min stirring, the suspension was centrifuged and washed several times with EtOH. The separated nanospheres were finally dried for 30 min at 950 °C. Figure 6 shows the scanning electron microscopy (SEM) images of the opal structure obtained with the vertical deposition of the pure SiO_2_ nanospheres, while the core-shell Er^3+^ doped nanospheres are shown in Figure 7.

The quality of the fabricated opals was investigated by means of transmission measurements: the stop band depth, i.e., the dip in percent transmittance at the stop band peak for normal incidence, was about 40% and the peak broadening Δλ/λc was 0.08, revealing good sample quality. The activated nanospheres were characterized by luminescence measurements: the typical photoluminescence spectrum of Er^3+^ was obtained at 514 and 980 nm, and a lifetime of 12.8 ms was measured. This value is close to the radiative lifetime for erbium in silica; hence, a quantum efficiency of 97% could be estimated [73].

The sol–gel route was also exploited for the fabrication of inverse silica opals, doped with RE ions, starting from a template constituted by polystyrene beads and infiltrating the direct opal with a sol; in a last step, the polystyrene nanoparticles were removed by thermal treatment. Large, well-ordered structures were obtained [74]; this system, doped with Er^3+^ ions, was characterized both optically and spectroscopically. The silica inverse opal exhibited a main emission peak at 1540 nm with a bandwidth of 21 nm and a lifetime of 18 ms, indicating a very high quantum efficiency. A similar structure, i.e., a silica inverse opal functionalized with a DNA-aptamer sequence labelled with Cy3 fluorophore was tested to develop a suitable platform for the realization of biosensors in a dye-labelled fluorescence detection scheme [75].

**Figure 8:**
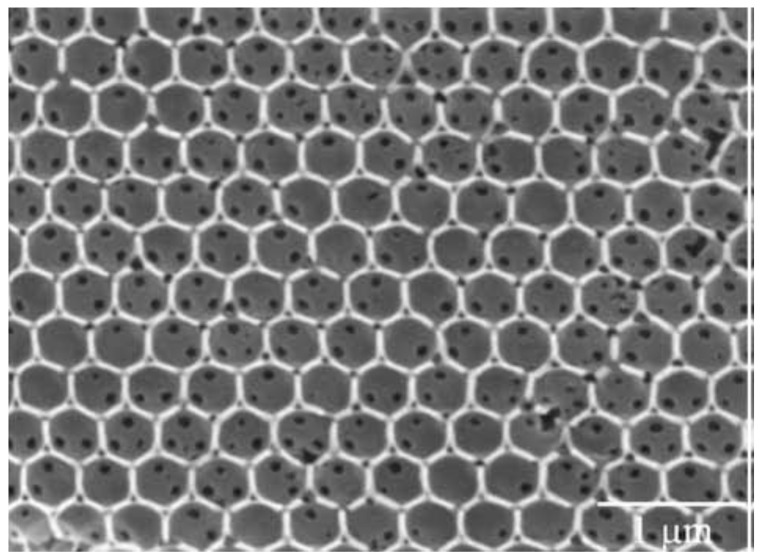
SEM micrograph of silica inverse opal structure, consisting of air spheres in a silica matrix. The template is based on 460 nm polystyrene sphere-derived photonic crystal made by convective self-assembly from a 0.1 wt% suspension in water. Reproduced with modification and permission from [76], copyright 2009, Elsevier.

Another example was presented by Goncalves et al. [76], who fabricated and studied from an optical and spectroscopic point of view a series of Er^3+^-Yb^3+^-doped inverse silica and titania opals. In more detail, the amount of Er^3+^ ions was varied between 0.25 and 1 mol% and that of Yb^3+^ ions from 1 to 2.5 mol%. The polystyrene direct opals, used as templates, were infiltrated by dip-coating and the inverse ones were obtained by calcination at 450 °C and, in some cases, at 900 °C. An image of the inverse opal structure, obtained using 460 nm polystyrene spheres, is shown in Figure 8.

### 4.2. Spherical and Bottle Microresonators

Whispering-gallery-mode (WGM) microresonators are an exciting application of pristine and rare-earth-doped glass spheres, usually with a size around one hundred microns. In a spherical or other circular-symmetry structure, optical rays are totally internal reflected and propagate along the surface as whispering-gallery modes [77,78]. If scattering losses and material absorption are low enough, these modes, that can be interpreted as electromagnetic waves circulating and strongly confined within the sphere, make the sphere operate as an optical resonator, which can possess a very-high-quality factor *Q*, even exceeding 10^8^ [79,80]. For this reason, the choice of the appropriate materials and, possibly, of a suitable coating film is of fundamental importance; again, sol–gel technology provides an effective tool.

**Figure 9:**
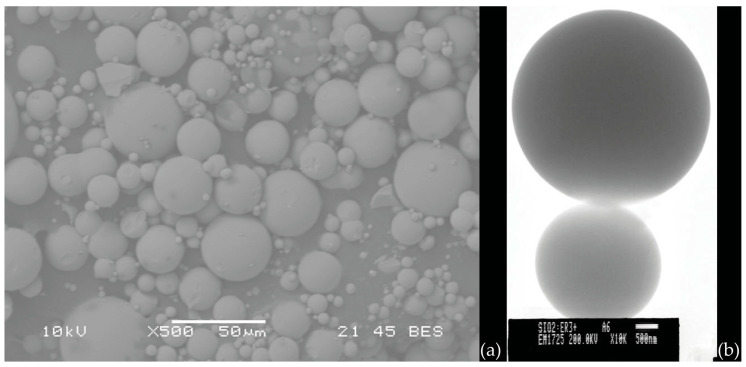
(**a**) SEM image of Er3+ silica spheres obtained by the acid catalyzed sol–gel route; (**b**) TEM image of two spheres demonstrating the high surface quality of the spheres. Reproduced from [82], under a Creative Commons license.

Very high-quality single microspheres are preferably made by melting the end of a pure silica (telecommunication grade) optical fiber; this technique, even if applicable also in the case of other glass fibers (e.g., chalcogenide fibers), greatly limits the choice of the material and may be complemented by other fabrication methods, such as the melting of a glass powder of the desired composition by using a plasma torch [81]. The simultaneous production of a great number of micro- and nanospheres in various types of glass is also possible thanks to sol–gel technology, as discussed in the early work by Righini et al. [82]. In that paper, the authors reported the synthesis of Er^3+^-doped spheres in acid conditions. The use of glacial acetic acid to catalyze the TEOS hydrolysis and polycondensation reactions caused the formation of highly polydisperse spheres, ranging from hundreds of nanometers to tens of microns; the smoothness of the surface of the spheres was extremely high. Briefly, a solution of TEOS:CH_3_COOH:H_2_O in the molar ratio of 1:4:4, with 1% Er^3+^ added, was vigorously stirred for 30 min at room temperature. After washing with EtOH and separation, the particles were dried at 80 °C overnight and then sintered at 950 °C and 1100 °C for 30 min. An example of the obtained spheres is illustrated in Figure 9.

**Figure 10:**
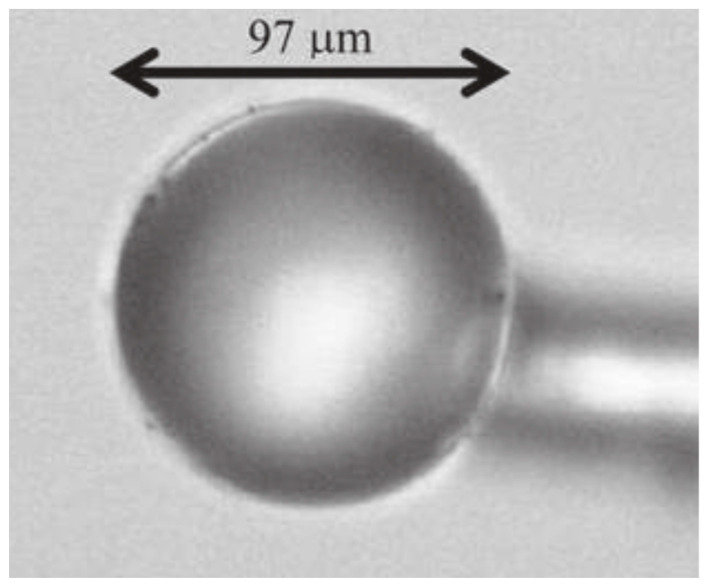
Optical micrograph of a single Er3+-activated silica sphere of about 100 μm diameter stuck onto a tapered silica fiber by means of a transparent optical component adhesive. Reproduced from [82] under a Creative Commons license.

The silica spheres could be separated and selected as a function of the size and then single spheres are stuck on a tapered fiber by means of an optical adhesive: a sample is shown in Figure 10. Among the various applications, on the basis of the optical and spectroscopic features of these structures, Righini et al. suggested the application of Er^3+^-doped spherical microresonators as microlasers operating at 1.5 μm. 

The properties of WGM microresonators are not unique to microspheres, but they are shared by other 3D and 2D structures with circular symmetry, such as hollow microspheres (microbubbles), microbottles, microrings and microdisks. Bottle microresonators are so-called because their profile often resembles an elongated spheroid or a microscopic bottle; they are often fabricated from an optical fiber by variation of its radius, but other techniques may be used, which include fiber annealing in SNAP (surface nanoscale axial photonics) technology, rolling of semiconductor bilayers, or solidifying a UV-curable adhesive [83].

**Figure 11:**
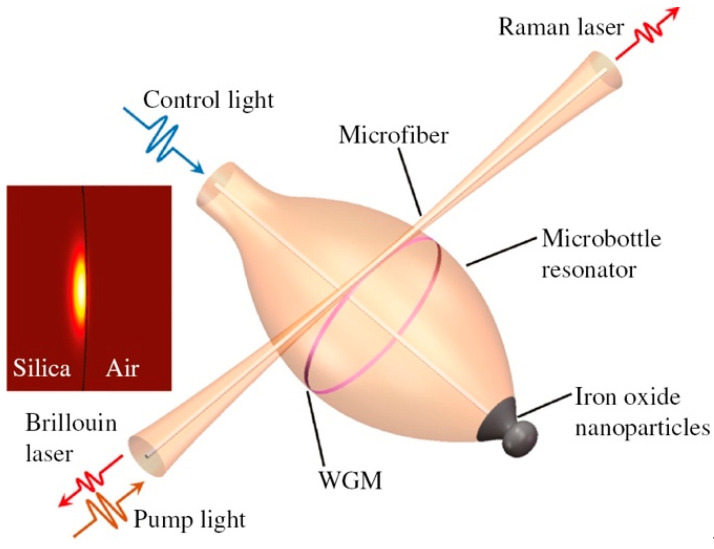
Sketch of the tunable Brillouin laser and Raman laser implemented in a hybrid microbottle resonator. The inset shows the fundamental mode field distribution of the resonator with a diameter of 114 μm. Reproduced from [86] under a Creative Commons license.

Microspheres, microbubbles and microbottles fabricated in RE-doped glasses represent an excellent tool for fabricating microlasers, due to their intrinsic ultrahigh quality factors (*Q*) and small mode volumes [84,85,86]. The injection of the pump light into a microresonator occurs through evanescent waves; for that purpose, tapered fibers are commonly used, which are fabricated by heating and properly stretching a standard single mode telecom fiber [77]. The coupling may be optimized by moving the tapered fiber closer to the sphere to have a good overlap of the evanescent fields of the fiber taper and of the resonator modes. Analyses of WGM modes, of coupling conditions and of microlaser operation have been reported in several papers [77,87,88,89,90]. Figure 11 shows the sketch of a hybrid microbottle resonator, coated with iron oxide nanoparticles on the tapered end, together with the coupling tapered silica fiber; the fiber injects the pump light (in the 1550 nm band) into the resonator and extracts the Raman or Brillouin laser signals. The quality factor Q of this resonator was over 10^8^ [86]. This microbottle was fabricated in a silica telecom fiber, but the use of a sol–gel coating offers the possibility of greater tunability and additional functionalities.

In fact, the use of a sol–gel coating on a microresonator, instead of a pristine glass microsphere, allows better tailoring of the modal characteristics of the resonator; the selection of specific radial-order propagation modes is possible when working on coating composition and thickness. By using a coating with a negative thermo-optic coefficient, it is also possible to compensate the thermal drift of a resonant frequency in the microresonator [91], or to tailor the free spectral range and geometrical cavity dispersion [92]. 

**Figure 12:**
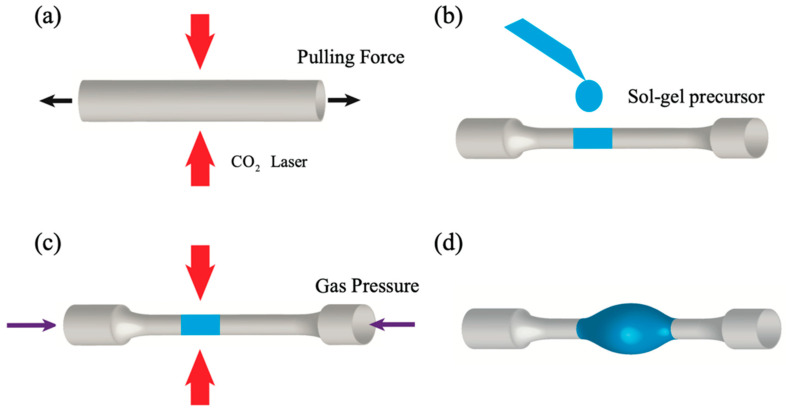
Fabrication process of a sol–gel-coated microbubble resonator using CO_2_ laser heating. (**a**) A capillary is tapered using CO_2_ laser heating and a pulling force. (**b**) Erbium-doped sol–gel precursor is drop-coated onto the tapered capillary. (**c**) Air pressure is applied inside the capillary while heating it by CO_2_ laser. (**d**) A sol–gel-coated microbubble is formed. Reproduced with permission from [93], copyright 2017, The Optical Society.

A microlaser was also demonstrated by using an Er^3+^-silica coating on a microbubble resonator [93]. The fabrication process is sketched in Figure 12: a silica capillary with outer diameter 350 µm and inner diameter 250 µm is heated by two counter-propagating CO_2_ laser beams and pulled until reduced to an outer diameter of around 30 µm. Then, a droplet of an Er^3+^ sol–gel precursor solution is transferred to the capillary (Figure 12b). By filling the capillary with compressed air and at the same time heating it again, a microbubble is formed. Due to the heating, the residual sol–gel solvent is removed, leaving only a silica film doped with erbium ions (maximum concentration ~5 × 10^19^/cm^3^). By injecting the 980 nm pump beam in the tapered coupling fiber (not present in figure), a laser emission at 1535.66 nm was observed, with a threshold estimated around 27 mW [93]. The peculiar characteristic of this laser is the possibility of tuning its emission with pressure; in fact, if the walls of the microbubble are thin, when one end of the capillary is sealed with epoxy and a compressed air cylinder is connected to the other end, an increase in the pressure makes the microbubble expand, so affecting the propagation modes. With a maximum applied pressure of 2.5 bar, the laser emission at 1535 nm was shifted by about 240 pm.

**Figure 13:**
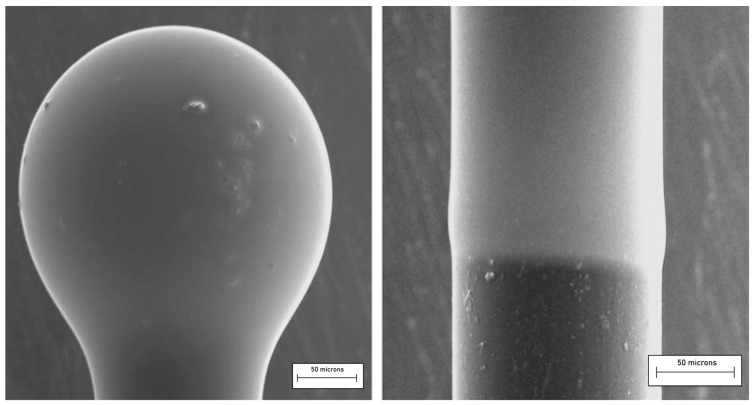
Micrograph of a silica microsphere coated with a 70SiO_2_–30HfO_2_ thin film and activated with erbium ions (**left**). The coating of a portion of the fiber stem (**right**) permits us to estimate the thickness of the deposited film. Reproduced with permission from [94], copyright 2009, Elsevier.

Similarly, microlasers may be realized by using sol–gel-derived RE-doped coatings, either amorphous or glass-ceramic, e.g., deposited onto a silica microspherical core obtained by melting the end of a standard telecommunication fiber SMF28. A near-perfect spherical shape with a diameter of hundreds of microns can be produced, and the photoluminescence functionality is added by depositing a proper sol–gel film by dip-coating. As an example, the protocol already developed to synthesize SiO_2_-HfO_2_ amorphous films was adopted to coat a microsphere [94]; the chosen concentrations were 70:30 as Si/Hf molar ratio and 0.1 to 1 mol% for the Er^3+^-doping level. The process allowed crack-free coating layers of about 1 μm thickness to be obtained, whose surface roughness was less than 2 nm. Figure 13 shows the SEM image of a silica microsphere, with a diameter of about 200 µm, coated with 0.8 µm silica-hafnia sol–gel film activated by Er^3+^ions. One can appreciate the good quality of the coating in terms of homogeneity and roughness. The observed defects are due to the dust particles present on the core’s surface.

A pump laser, usually at 980 nm, may then be coupled to the microresonator and absorbed by Er^3+^ ions that emit in the C-band around 1550 nm. The same approach may be followed in the case of microbottle and microbubble resonators [93].

Ristic et al. [95,96] studied the effect of the increase in thickness of a 70SiO_2_–30HfO_2_:0.3 Er^3+^ (mol%) coating on the WGM luminescence intensity as well as on the coupling efficiency. The authors observed that the luminescence intensity increased linearly with the coating thickness and that the effect of coupling on the luminescence-intensity behavior was different for different excited WGMs. A very important conclusion of their study was that in order to couple light in and out of a microsphere, the refractive index of the microsphere is not very critical for achieving efficient coupling. Because of the high number of azimuthal modes, in fact, it is always possible to achieve phase-matching to one or more of these modes regardless of the sphere refractive index. This result was relevant since it means that for the practical applications of sol–gel coated microspheres, e.g., for micro-lasers or sensors, there is a high degree of freedom in the choice of the sphere and coating materials as well as in the coupling scheme, making it much easier to construct efficient devices.

### 4.3. One-Dimensional (1D) Microcavities

Another area of photonic structures, besides opal and circular-symmetry resonators, where sol–gel technology has proven to be crucial is the fabrication of 1D microcavities. One-dimensional photonic crystals exhibit a photonic bandgap that can be tailored to enhance the luminescence quantum yield and in general to manage the spectroscopic properties exploiting the photon confinement. 

**Figure 14:**
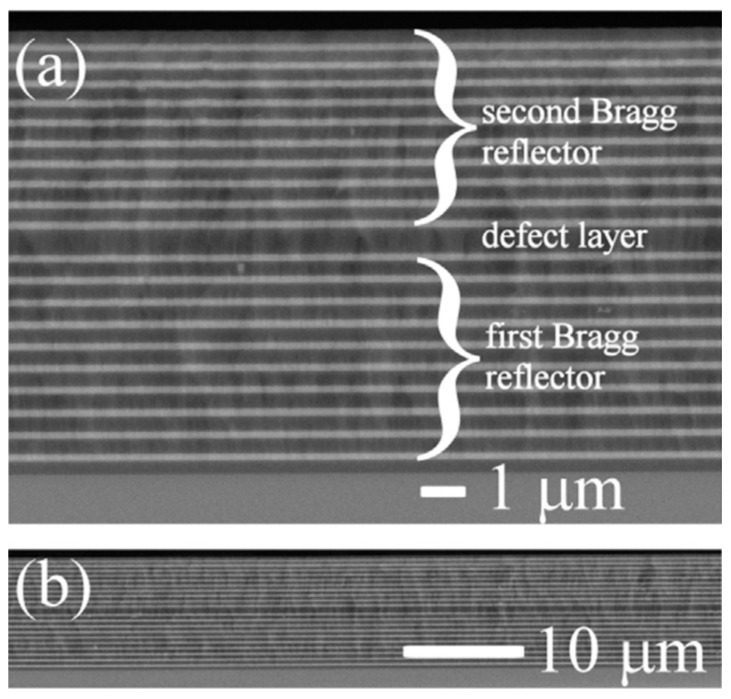
SEM image of the cross section of a 1D microcavity fabricated by Chiasera et al. [99]. The bright and the dark areas correspond to TiO_2_ and SiO_2_ layers, respectively. The substrate is located at the bottom of the images and the air at the top. (**a**) Image of a section of the sample of about 16 µm in length. (**b**) Image of a section about 60 µm long. Reproduced with permission from [99] Copyright 2015, Elsevier.

Almeida et al. [97] discussed the fabrication and characterization of RE-doped photonic crystal microcavities prepared by sol–gel. They realized microcavities constituted by Er^3^-doped or Er^3+^/Yb^3+^-co-doped active SiO_2_ layers placed between distributed Bragg reflectors consisting of three alternating SiO_2_/TiO_2_ pairs. They found an enhancement of the Er^3+^ spontaneous-emission intensity by a factor of up to 18 when inserted into the microcavity and a strong sensitizing effect (sensitizing factor 25) in the case of co-doped samples upon excitation at 980 nm. An alternative configuration was proposed by Jasieniak et al. [98] who developed a sol–gel-based vertical optical microcavity with a quantum dot defect layer. The asymmetric Bragg microcavity was constituted by a Bragg reflector and a metal mirror to confine the light in the defect layer. The choice of such a structure was motivated by the necessity to avoid photoluminescence quenching caused by the quantum dot damage induced by the high temperatures needed to process the second sol–gel-based Bragg reflector. A very effective hybrid dielectric microcavity was presented by Chiasera et al. [99], who used a hybrid strategy to overcome the problem of the high temperature process. In fact, Bragg reflector layers were deposited via radio frequency sputtering and the structure was tailored in order to obtain the resonance centered at about 630 nm. First, two silica glass substrates were placed in the vacuum chamber to obtain two identical Bragg reflectors. Both Bragg mirrors consisted of 20 alternating layers of silica and titania, with a titania layer last, defining the interface sample/air. Figure 14 shows a SEM micrograph of the cross section of a fabricated 1D microcavity [99]; the two images correspond to a section of the sample about 16 µm long (a) and a section about 60 µm long (b). Figure 14b makes evident the good thickness uniformity as well as the perfect adhesion of the films over a rather long extent. An active layer was made out of a poly-laurylmethacrylate matrix embedding CdSe@Cd_0.5_Zn_0.5_S quantum dots, which acted as the emitters, and it was deposited on one of the Bragg mirrors. Finally, the active layer came out sandwiched between the two mirrors and subjected to thermal annealing. A quality factor of about 890 was measured for this cavity. The effect of the cavity on the ^4^I_13/2_→^4^I_15/2_ emission band was demonstrated by the narrowing of the emission band as well as by the enhancement of the Er^3+^-photoluminescence intensity. The coherent character of the emission was discussed, and then proved in a later paper [100].

A completely different application of sol–gel-TiO_2_ films was proposed by Prusakova et al. [101], who produced and characterized, from a morphologic, structural and optical point of view, very thin films (few nm thick) for memristive devices. For the sol synthesis, 1 mL of ethanolamine, 10 mL of 2-methoxyethanol and 2 mL of titanium isopropoxide were stirred in a round bottom flask equipped with a reverse condenser in dry N_2_ atmosphere for 2 h at 80 °C and 1 h at 120 °C. The resulting dark-purple sol was cooled down and diluted with EtOH or 2-propanol in a 1:2 or 1:3 ratio using a dry Schlenk’s flask. The sol was then filtered through a 0.2 μm syringe filter and spin-coated on fused silica quartz substrates patterned with a Ti (5 nm)/Pt (50 nm) layer. Spinning was carried out in different steps: first, at 1300 rpm for 2 s, followed by 2000 rpm for 2 s and finally 3000 rpm for 49 s. After the deposition, the samples were dried at room temperature for 20 min, then preheated for 1 h at 150 °C. Two-layer samples were also prepared, repeating the same procedure. All the samples were cured at 150 °C in air for 1 h and annealed at 400 °C for 1 h, to eliminate residual porosity; the heating/cooling rate was 1 °C/min. MicroRaman measurements indicated the crystallization of titania in the anatase phase, in agreement with observations made by field emission scanning electron microscopy (FE-SEM). Preliminary electrical characterization of the samples suggested that the TiO_2_ films fabricated by this process are potentially useful for the realization of memristive systems.

Titanium dioxide, on the other hand, is a well-studied material for many other applications and, recently, growing attention has been devoted to the engineering of its bandgap [102], which depends on its phases (amorphous or crystalline: anatase, rutile, and brookite) and plays an important role in photonic devices and solar cells. Furthermore, the cobalt-doping of sol–gel TiO_2_ nanostructures has been shown to have interesting applied perspectives, especially in the field of spintronic and magneto-optic devices [103].

Another important issue concerns the use of the sol–gel process to fabricate transparent glass-ceramics, which are employed in many fields. Glass-ceramics [104] combine interesting properties of both amorphous and crystalline phases and offer specific characteristics of capital importance in photonics. By engineering the glass-ceramics chemistry, the nature, or volume fractions of crystalline and amorphous phases, several interesting properties related to the RE-doped luminescent nanocrystals (fluorides, chlorides, oxychlorides, etc.) can be achieved and tailored so that the sol–gel technique appears to be one of the most versatile processes for the fabrication of photonic systems [105,106,107,108,109].

## 5. Conclusions

The sol–gel method is an efficient and flexible alternative technique for glass synthesis with respect to melt quenching, especially to produce materials doped with photoluminescent ions with higher purity and homogeneity. Moreover, it allows the fabrication of photonic structures in different shapes such as bulk, fiber and thin film and even in non-conventional forms. 

The aim of this short review was to highlight some basic issues that are mandatory for the development of photonic structures based on sol–gel technology. We have presented some consolidated arguments mentioning papers from the onset years of glass photonics. The main topics concerning the role of composition and fabrication protocols have been discussed, making reference to published research papers that were a direct output of our activity about these crucial points. Among other interesting matters, we underlined the role of OH groups on the quenching of the luminescence and the different roles of the rare-earth ions acting as “glass modifiers”. The use of specific optical transitions to assess luminescence quenching was also presented. Some examples of micro or nano photonic structures, where the role of the sol–gel method is crucial, have also been shown, including 3D photonic crystals, 1D microcavities for low-threshold laser action, WGM microresonators, and memristors.

We recognize that we have reviewed only a narrow portion of the relevant experiments and achievements in the field of sol–gel photonics, and for that we apologize to the readers. On the other hand, research in this area has been ongoing for almost 40 years and it would have been impossible to be exhaustive; we only hope that these few pages could stimulate the reader’s curiosity and could lead him/her to explore the vast territory of sol–gel photonics and its applications in other fields, such as protective coatings, solar energy, sensors, and bioglasses, to mention just a few of them. In all these areas, sol–gel technology still has much to offer to brilliant researchers. 

## Data Availability

For figures previously published, the necessary permissions to publish have been obtained from the copyright holder.
